# Workplace Mobbing in Polish and Lithuanian Organisations with Regard to Corporate Social Responsibility

**DOI:** 10.3390/ijerph17082944

**Published:** 2020-04-24

**Authors:** Jolita Vveinhardt, Włodzimierz Sroka

**Affiliations:** Management Department, WSB University, Cieplaka 1c, 41-300 Dąbrowa Górnicza, Poland; jvveinhardt@wsb.edu.pl

**Keywords:** workplace mobbing, CSR, Poland, Lithuania

## Abstract

The ‘mobbing’ phenomenon is regarded as the actions or behaviour referring to an employee or directed against an employee, comprising persistent and drawn-out harassment or intimidation of that employee. This phenomenon causes substantial negative workplace consequences, but, above all, one should stress the consequences for the victims, which are devastating. This has been observed in a variety of organisations, regardless of the sector and country. Given these facts, the purpose of this study was to identify the prevalence of workplace mobbing in Polish and Lithuanian organisations with regard to corporate social responsibility (CSR). The research sample included a group of 823 entities operating in both countries in both the private and public sectors (410 from Lithuania and 413 from Poland). A closed-type questionnaire was used in the survey. Several research methods including factor analysis, Cronbach’s alpha, Spearman–Brown, factor loading, and total item correlation were used in our study. The results achieved showed that there were both similarities as well as differences between the analysed organisations. More specifically, our research revealed that: (1) Employee attitude to CSR depends on the company’s sector of activity and the country; (2) In Poland, workplace mobbing is more prevalent in the public sector than in the private, whilst in Lithuania there were no substantial differences; (3) Organisations that implemented the CSR concept showed less imposed mobbing prevalence; and (4) Employees who faced mobbing in the workplace had worse relationships with clients and users of the company’s services/products.

## 1. Introduction

How does the declaration of corporate social responsibility (CSR) influence employee interrelationships and how does the existence of workplace mobbing in an organisation affect the very employees’ relation to CSR? Is CSR a guarantee that workplace mobbing will be eliminated? These and similar issues that have arisen from organisational practice show the considerable interest of organisations in external stakeholder and organisational image [1,2]. A particularly significant imbalance between the focus on external stakeholders and the reconciliation of internal processes occurs both in old market economies [3,4,5], and in the Middle and Eastern European countries that broke free three decades ago that have put greater emphasis on economic responsibility or environmental protection than on social aspects [6]. Here, the engagement of different countries and practices differ [7]. It is likely that in combining shareholders’ interests and public expectations, organisations are looking for a simpler way to improve the opinions of external stakeholders, but less noticeable are internal processes that determine not only employee support for the CSR program, but also the well-being of the very employees of the organisation, which remain in the background.

From a theoretical standpoint, significant links between CSR goals and employee protection from workplace mobbing can be found. Solutions of the problem of workplace mobbing as a psychosocial stressor are intended for the creation of a healthy and safe environment [8,9,10,11], and employee health and psychosocial well-being are constituents of the CSR concept [12,13,14,15] that have a positive impact on employee satisfaction and organisational performance [16]. Although there is evidence that employee engagement significantly contributes to CSR and increases the productivity of their own activities [17], it has, however, also been noted that there is insufficient research on how employees perceive social responsibility within their organisations [18] and on the inclusion of employee well-being in the CSR policy [19,20]. Furthermore, the tendency is observed to shift social responsibility to “others”, and to disassociate from it [21,22].

These observations are important because the way employees accept social responsibility values may determine their own relation not only with the external, but also with the internal environment, that is, with co-workers [23]. For example, although there are also opponents of the CSR concept (e.g., [24]), some studies have shown that CSR plays a positive role in building communal relationships between the organisation and its employees [25], stimulates positive emotions in employees [26], and is generally associated with a more favourable psychological climate inside the organisation [3]. Conversely, troubles in organising internal processes turn into a factor provoking workplace mobbing or creating conditions for its escalation [27,28,29,30,31,32]. Therefore, it could be predicted that inclusion of social responsibility in the organisational agenda should be of service to the greater well-being of employees and reduce the opportunities of such dangerous phenomenon as workplace mobbing. However, research shows that even CSR standards themselves depend on the context of the country in which the organisation operates [33,34], besides, there may be difficulties when organisations lack policy for psychosocial risk management [35]. On the other hand, although both CSR and workplace mobbing problems have separately received considerable attention from researchers, there is a lack of research dealing with the prevalence of mobbing depending on the status of the organisation in the context of CSR, and how this relates to the social responsibility of the employees themselves. In addition, it makes sense to compare countries that are culturally and historically close. Given these facts, the purpose of our paper was to identify the prevalence of workplace mobbing in Polish and Lithuanian organisations with regard to corporate social responsibility.

The paper is organised in the following sections: the literature review is presented in Section 2 and Section 3 highlights the data and methodology used in our research. Section 4 presents the main findings of the paper and discusses the results. Finally, Section 5 summarises the conclusions of the study.

## 2. Literature Review

Mobbing is regarded as one of the most serious psycho-social hazards in the work environment. One should state that researchers and practitioners from different disciplines including organisation and management, or psychology, sociology, and law have not ceased in their efforts to analyse this threatening phenomenon to find ways to reduce it and create a safe working environment [36]. Mobbing is a heterogeneous phenomenon, so it is necessary to look at the different approaches. Leymann [37] (p. 167) described mobbing as “social interaction through which one individual (seldom more) is attacked by one or more (seldom more than four) individuals almost on a daily basis and for periods of many months, bringing the person into an almost helpless position with potentially high risk of expulsion”. Though there are various reasons for the existence of mobbing, Paparella et al. [38] claim that a particular one is the increase in fixed-term employment forms. As the result, companies may recruit new employees more flexibly and t select those who meet their needs to the highest extent. Such strategies are often adopted by individual managers without the agreement of the company. However, they have a negative impact on the victims facing such behaviour, and leads to isolation, guilty feelings, and even to resignation. It can be said that mobbing is the result of the lack of a sympathetic climate among colleagues, especially if this is fuelled by the individualism of some of them.

The costs of mobbing at the workplace are substantial (if not even higher), but above all, one should stress the consequences for the victims, which are devastating. In addition, aside from image deterioration, it has a substantial impact on the financial situation of the companies in which mobbing occurs. An evaluation made by the International Labour Office estimated that a company that employs around 1000 employees bears the costs of psychological harassment at the level of EUR 150,000 a year. One should also stress the reduced working performance of the victims of mobbing (by 60%) [38].

Mobbing is a phenomenon that crosses national borders as well as sectors. One may ask the question whether there is a sector that is free of this phenomenon, and the answer would probably be ‘no’. This phenomenon has been observed in almost all companies regardless of their size (small, middle size, large and global companies) as well as sector of activity (public and private; industrial vs. service). A number of studies have confirmed this fact. Akyüz et al. [39] analysed the profile of mobbing in the forest products industry in Turkey where their research concentrated mainly on analysing the relation of mobbing with organisation. The results showed that the frequency of mobbing in the aforementioned sector was found to be 21.67%. What was interesting is that employees working for 6–10 years at the same enterprise faced mobbing much more than the others. Using the quantitative research approach, Sandybaev [40] conducted research on the hospitality sector in Northern Cyprus. Here, 360 participants were assessed by questionnaire. It may be shocking that as much as 41.9% of all respondents perceived themselves as victims of mobbing. However, practically, this number was higher as participants who indicated themselves as non-victims also reported that they were exposed to many forms of mobbing behaviour in their workplace. In other words, the results clearly demonstrated that mobbing within the sample groups is widespread and this fact has individual consequences.

Yildirim and Yildirim [41] analysed the mobbing phenomenon experienced by nurses working in healthcare facilities in Turkey. In total, there were 505 participants of the research, of whom 325 (64%) worked in public and 180 (36%) in private hospitals. All of the participants were female. The overwhelming majority (86.5%) of the nurses participating in the research reported facing mobbing behaviour in the workplace in the previous year. Furthermore, nurses working at private hospitals faced statistically significantly more mobbing behaviours than those at public hospitals (*p* ≤ 0.02). The nurses who faced mobbing behaviours presented a variety of psychological and emotional reactions to these incidents. For example, 10% of the respondents stated that they ‘considered committing suicide sometimes’. Similar results were indicated in other surveys conducted by Ovayolu et al. [42] amongst 260 nurses working in three public hospitals. The majority of nurses were female and reported being assigned duties outside their usual responsibilities, held responsible for coworkers’ mistakes, and were criticised for job performance even though they thought they had done their work properly. Most of the nurses experienced health and sleep problems as well communication problems with other staff members. All these effects brought about a situation where many nurses have sought psychological support to solve their problems.

Finally, Erdogan and Yildirim [43] conducted research amongst 479 healthcare professionals representing different positions (i.e., doctor, nurse, physiotherapist, dietician, psychologist, and social service worker) who provided services in five state owned hospitals in Turkey. It was not surprising that like the previous research in this sector, the mobbing phenomenon was also confirmed. It was revealed that the rate of exposure to one of the sub-dimensions of mobbing scale at least once in the last year was 66.4% for isolation, 71.8% for attack on professional status, 78.1% for attack on personality, and 28.4% for direct negative behaviours. Females as well as participants with low income were much more exposed to mobbing behaviours. For example, nurses, when compared with doctors, were much more exposed to mobbing. Moreover, individuals with an occupational experience of >10 years were more exposed to mobbing. No wonder that these phenomena have caused a number of negative consequences for the victims (e.g., lower job satisfaction and isolation).

The deliberations presented allow us to state that, regardless of the country, workplace physical mobbing against healthcare professionals is a widely observed phenomenon. However, Li et al. [44] also mentioned another problem that exists in the sector (i.e., violence perpetrated by patients and visitors). Their research showed that this has also been a persistent problem worldwide.

Other findings were obtained by Kum and Ertas [45], who analysed the Turkish maritime industry (ship, shipyard, public and private company workers). According to their results, employees were rarely exposed to mobbing (mean: 1.85, standard deviation: 0.42, minimum: 1.13, maximum: 2.83). This means that the level of mobbing in the Turkish maritime industry was very low and rare. It is assumed that there are two main reasons for this result: participants were either not feeling free to reply to such questions (another kind of mobbing) or they did not recognize this concept.

Before analysis of the literature, one might predict that the mobbing phenomenon is usually a characteristic feature of less developed countries, and the deliberations presented so far might confirm this thesis. However, when analysing the situations observed in well-developed countries, we observed the prevalence of the same phenomena. Research on mobbing prevalence carried out in EU countries by Giaccone and di Nunzi [46] highlighted some interesting results. They revealed that the Baltic states, Central and Western European countries as well as Scandinavian countries were above the EU 28 average of 14%. Furthermore, Austria, the Czech Republic, and Finland showed the highest percentages of workers reporting violence or harassment at the workplace (more than 20%), whereas in half of the Eastern European countries (except Slovakia, Slovenia, and the Baltic states) and in all of the southern European countries, a smaller proportion of workers reported adverse social behaviour (ASB), from 6% in Cyprus to 12% in Croatia [46].

Equally interesting is a more detailed analysis of the situation observed in particular EU countries. For example, in 1998, the European Foundation for the Improvement of the Living and Working Conditions estimated that in Italy, about 4.2% of all workers were victims of mobbing. Moreover, according to data compiled by national experts in 2004, the proportion varied between 4% and 6% of the total work force (i.e., between 1 million and 1.5 million workers [38]).

In addition, there are significant differences between the victims of mobbing in particular countries. Such differences were especially visible in Italy. These results were confirmed, inter alia, by the study conducted by the Bocconi University of Milan on a sample of 1000 people chosen among 3000 victims who consulted the Milan Labour Health Clinic (Clinica del Lavoro). The study also revealed that in Italy, victims were almost equally divided among women (51%) and men (49%), while in the rest of Europe, the clear majority of victims were women. Moreover, there is an important difference in the way mobbing is displayed in the public and private sector: in the public sector, there is more gossip, intrusion into private life, and spread of false information to put the worker in a situation of psychological unease; while in the private sector, there is more continuing harassment, increases in workload, and isolation to induce the worker to resign [38].

Maran et al. [47] analysed the mobbing phenomenon using Italian civil court judgments in the period of the last fifteen years (based on the data collected from two free websites on civil judgments involving mobbing). Herein, of the 73 civil judgments, 34 victims of mobbing were male and 39 victims were female. While the total number of cases was not shocking, the fact that they constituted 46.6% (male) and 53.4% (female) victims, respectively, is. In addition, in 68 cases, the behaviour that characterised the mobbing campaign was an attack on the victim’s personal and professional life. Female victims of mobbing in particular indicated isolation and attack on reputation. As stated by Galletta et al. [48], the impact of mobbing on people can be devastating and can produce several psychological and physical symptoms. In turn, Monaco et al. [49] analysed the situation in Sant’Andrea Hospital (Rome, Italy) in the period between 2001 and 2012. According to the collected data, in total, 814 users were certified for compatibility bullying (63% of all cases) and showed that mobbing is as a real “organisational pathology”.

Similar data were achieved by Stanisławska et al. [50], who conducted research in Poland in the West Pomeranian Voivodeship within a group of 418 people aged 19–64 who represented three working environments: civil court officials (30.86%), employees in the healthcare sector (49.76%), and supermarket chain employees (19.38%). The analysis of the intensity of the phenomenon of mobbing in relation to the workplace showed statistically significant differences (*p* < 0.05). The highest average mobbing intensity was 2.43 for supermarket employees, the average for healthcare professionals was 1.36, and the lowest average was 0.51 for court employees. The greatest intensity and exposure to mobbing occurred among supermarket employees. In healthcare, the phenomenon of mobbing more often affected people with more seniority.

Furthermore, Zacharová and Bartošovič [51] analysed healthcare service (nurses), but in the Moravian-Silesian region in the Czech Republic. Their research sample involved a total of 113 participants. Accordingly, 31 (27%) of all nurses who participated in the research had faced mobbing behaviours in the last six months. The most serious problem mentioned by the respondents was ‘attack to personality’ and the most common reactions to escape from mobbing were ‘to work more carefully to avoid criticism’ and ‘to work harder and more organised’. The negative consequences of mobbing behaviours included feeling tired and stressed, headaches, and irregular sleep.

In light of the deliberations presented, the case study analysis based on Arcelor Mittal Poland, being part of the Arcelor Mittal Steel group (no. 1 in the world steel industry), practices sound interesting. The interviews indicated that only single cases of mobbing and harassment activities (though relatively rare) were observed. Generally, however, almost everybody had heard about some cases of mobbing (it is possible that they were related to the same incidents) and these mobbing activities were distributed among three groups of employees: (1) those who did physical work (are directly engaged in the production process); (2) employees involved in the administrative activity and sales; and (3) managerial staff. According to the managers interviewed, such a result may be due to the decreasing number of employees, especially in the production sphere, and with responsibilities being merged [52]. Based on the deliberations presented, the following hypotheses were formulated:

**Hypothesis** **1** **(H1).** *Employee attitude to CSR depends on the company’s sector of activity and the country.*

**Hypothesis** **2** **(H2).** *Workplace mobbing is more prevalent in the public sector than in the private one.*

In the literature, a large scientific discussion exists on the direct or indirect benefits of enterprises being socially responsible (e.g., [53,54]. One claims that companies should not only create profits, but should also act as subjects that enhance sustainable development and responsible behaviour [55]. There is a general acceptance of the thesis that if a company wishes to be perceived as a reliable partner in business, it should behave in accordance with CSR concepts, implement elements of this concept, and, indeed, follow this concept fully [24,56]. In general, this means that it should behave responsibly [57]. This ethical behaviour of enterprises is based on the principles of honesty, integrity, and trustworthiness [58]. One can then state that the prevalence of mobbing in the organisations is connected with the fact of whether they are socially responsible. This implies that a company that operates ethically will not indulge in mobbing, and vice versa. This means that if manifestations of mobbing are observed in organisations, such organisations will not be perceived as ones that apply ethical behaviour guidelines. Thus, the CSR concept can be used to prevent mobbing, and the creation and implementation of effective anti-mobbing programs, put into practice as part of this concept, reduces the occurrence of this phenomenon [59]. This outcome was especially visible in the case of Arcelor Mittal Poland, which realizes the idea of “a responsible company” through supporting local society in the spheres of culture and art, education, health, ecology, sport, and employee volunteering [52]. The number of cases of mobbing therein was rare. Still, the examples presented do not change the fact that there is still insufficient research that directly deals with mobbing in the context of CSR. Based on this fact we formulated hypothesis H3.

**Hypothesis** **3** **(H3).** *Mobbing prevalence is less in organisations that implement the CSR concept.*

One of the tasks of HRM is the creation of an atmosphere in the workplace that will ensure that employees are able to use their skills and knowledge as effectively as possible [60]. In practice, however, this task in not easy and a number of surveys have presented the negative consequences of mobbing in the workplace. Additionally, a number of surveys have been devoted to the negative consequences of mobbing in the workplace, and researchers have underlined a variety of factors. For example, exposure to mobbing can lead to heightened job insecurity and intention to leave as well as a lack of job satisfaction [61,62], sleep disturbances [63,64], some psychological problems such as anxiety, fatigue, helplessness, and depression [65,66], burnout [50,67], lower self-esteem [68] and post-traumatic stress disorder [47], less organisational citizenship, and more counterproductive work behaviour [69]. Companies where mobbing exists face absenteeism, turnover, and replacement costs, lowered productivity and performance as well as grievance procedures, not to mention the loss of public reputation and image [70]. As some studies have shown, social responsibility positively affects attitudes and the loyalty of the company’s stakeholders (e.g., product users and employees [71,72]), there is no doubt that the employees who are imposed on by mobbing will be less inclined to behave ‘normally’ to their clients, thus having worse relationships with them. This fact has allowed us to formulate hypothesis H4:

**Hypothesis** **4** **(H4).** *Employees who face mobbing in the workplace have worse relationships with customers and users of the company’s services/products.*

## 3. Materials and Methods

Two countries (i.e., Poland and Lithuania) were chosen for a comparison. Both countries are neighbouring, and are characterised by similarities (like cultural heritage, similar development level, GDP per capita) as well as by substantial differences. For example, Poland is five times bigger geographically with a population of 38.5 million people, while Lithuania only has around three million people [73]. The research instrument developed for revealing trends regarding workplace mobbing in Polish and Lithuanian organisations with regard to corporate social responsibility was based on the items provided in the questionnaire entitled “Mobbing as a Psychosocial Stressor in the Organisations Accessing and Implementing Corporate Social Responsibility–MOB-CSR” [74,75]. The questionnaire was adapted through preparing it in both Polish and English languages. Adaptation took place in several phases:

― In the first phase, two independent translators who did not know each other translated the questionnaire into Polish and two translators into English.
― In the second phase, the synthesis of two translations into Polish and two into English was performed.
― In the third phase, to assess the synthesis of the translations to both languages, the first expert assessment involved three experts holding PhDs in the social sciences (the field of science: management, psychology; areas of research interests: workplace mobbing, corporate social responsibility) was performed.
― In the fourth phase, the authors made wording corrections to the statements, considering the experts’ average score (from 1 to 5) and the comments made.
― In the fifth phase, the survey of the target population, attended by six respondents, was conducted. Of these, three respondents were native speakers of Polish and three were native speakers of English.
― In the sixth phase, after testing with the target population, the wordings of the statements were adjusted.
― In the seventh stage, two translators who did not participate in the second phase did a back translation of the questionnaire.
― In the eighth stage, the questionnaire was reviewed after back translations, minor corrections were made, and the final version of the questionnaire intended for distribution was prepared.

The statements of the questionnaire categories revealing factors related to workplace mobbing were coded negatively, while the factors related to corporate social responsibility were recoded. Prior to conducting the study, study participants were given an explanation of the study being conducted, what the aim of the study was, how the data collected during the study would be protected, and where the study results would be used (i.e., depersonalised, generalised results would be published). Respondents were also informed that they could terminate their participation in the study at any time, withdraw their consent, and choose not to answer questions should they choose to do so. Confidentiality and anonymity were guaranteed; it was explained to the participants as to how the collected information would be destroyed after data processing. The questionnaire survey was initiated only after ascertaining that the respondents understood their rights and did not object either in words or actions. Study participants voluntarily participated in the study without receiving any remuneration.

The questionnaire consisted of 113 items in total. Statistical verification of the validity and reliability of the questionnaire during the exploratory research showed its high psychometric characteristics. In a sample of this, research validity and reliability tests were conducted using factor analysis and explained dispersion (%), Cronbach’s alpha, Spearman–Brown, factor loading (L), and total item correlation (r/itt) (Table 1 and Table 2).

The research sample involved 813 respondents employed in Lithuania (hereinafter LT) and Poland (hereinafter PL) (*N* = 410 LT and *N* = 413 PL). The sample of the research was sufficient for verification of the questionnaire, according to a graded scale of sample sizes for scale development, proposed by Comrey and Lee [76]: when a sample is 300 respondents, the sample is considered good; when a sample is 500, very good. In this case, our sample belonged to the category ‘very good’. The survey was conducted electronically, meaning that the questionnaire was placed on an electronic survey platform. The survey platform apklausa.lt was used. The questionnaire was not available to the public (i.e., only the organisations to which electronic survey links were sent could complete the questionnaire). The online survey platform also had a prohibition function enabled, which prevented someone from filling in the questionnaire for the second time from the same computer. As the submission of the questionnaire was impossible without the full completion of the questionnaire, no questionnaires were rejected because of incompletion. The possibility of the same ratings in a subcategory was also forbidden, thus, in this case, all questionnaires were also properly completed.

The hypotheses were tested using the Mann–Whitney U test, Kruskal–Wallis H test, and Chi-Square test.

## 4. Results and Discussion

As mentioned earlier, the survey involved a total of 823 respondents. The survey was conducted in Lithuanian and Polish. Table 1, Table 2 and Table 3 present the results of the psychometric characteristics and prime and secondary factorisation of the questionnaire for the sample *N* = 823.

The results given in Table 1 show that the explained dispersion percentage (the lowest for all subcategories was 51.08; the highest, 84.06), Cronbach’s alpha coefficient (the lowest was 0.75; the highest, 0.95), factor loading (L) (the lowest minimum factor loading was 0.48; the highest minimum factor loading was 0.92), the total item correlation (r/itt) (the lowest mean was 0.48; the highest mean, 0.84) met the requirements for the questionnaires even in the case of the lowest values obtained. In this case, the Spearman–Brown coefficient was not calculated in those subcategories, which included four statements and less than four statements, and this coefficient shows the category’s internal consistency, internal compatibility, and reliability, but is calculated using a method other than Cronbach’s alpha and is not affected by the number of statements in the subcategory.

Since this questionnaire contained 113 statements, it is particularly important to perform prime and secondary factorisation, which is calculated for large-scale (as in this case) questionnaires. Table 2 and Table 3 present the results of the prime and secondary factorisation at both the level of categories and subcategories (Table 2) and at the level of parts of the questionnaire (Table 3). Subcategories comprising a certain category must have a similar meaning. During prime factorisation, the set of criteria is calculated, while during secondary factorisation, the criteria calculated are combined into categories. The closer the factor loading is to 1, the more the individual statement of the questionnaire corresponds to the distinguished factor. The results at the level of six categories and 15 sub-categories of the questionnaire scored the highest percentage of respondents’ approval (according to the principal component method) of 87.85% and relatively the lowest (i.e., 64.99%). The results of the factor analysis for two parts of the questionnaire showed that the percentage of the explained dispersion was very similar. Thus, the results of the secondary factorisation only reaffirmed the high methodological characteristics of the categories and sub-categories of the questionnaire.

The characteristics of the respondents involved in the research and the organisations that they represent are presented in Table 4.

No statistically significant differences (*p* > 0.05) between public and private sector enterprises in Lithuania were recorded either in the context of mobbing factors or socially responsible behaviour. This shows that the same forms of malicious behaviour with co-workers were found in both the public and private sector and that employees’ reactions expressed as their intention to leave the workplace in principal did not differ. It can be seen that the aspects of corporate social responsibility applied to a greater or lesser degree in both sectors. Still, there was no significant difference in employee behaviour with regard to CSR (Table 5).

Business enterprises in Poland are more oriented to the socially responsible behaviour of the organisation and its employees, while public sector organisations encounter mobbing problems more often. Although a significantly worse status of employee interrelationships was identified when compared with the private sector, the situation related to organisational decision-making at the management level (nature of tasks, work content and assessment, working conditions) came to prominence most often. This might have also determined the more pronounced public sector employees’ intentions to change jobs. From the standpoint of socially responsible activity, the situation in the public sector was significantly worse. In this sector, less attention was paid to services and their quality as well as to customer information, health, and security. Aside from the aforementioned, a statistically significant difference in employee attitudes was also noticeable; that is, representatives of the public sector demonstrated lower social responsibility (Table 6).

The data presented in Table 4, Table 5 and Table 6 allow us to state that hypothesis H1 was confirmed in both countries.

In turn, H2 was confirmed only in Poland. It may then be stated that despite many similarities between both countries, there were also substantial differences. This confirms the findings of Kliestikova and Janoskova [77], who analysed the profiles of consumers in different countries. They revealed, inter alia, that despite the fact that the Slovak Republic and Czech Republic have a common socio-cultural past due to existence of the former Czechoslovakia, they were grouped into different clusters.

Problems of employee interrelationships in all aspects and problems related to tasks, work content, and assessment in almost all aspects were more often encountered in Polish public and private sector organisations, where estimates statistically significantly diverged when compared with Lithuanian organisations. The intentions of the respondents of both states did not differ only in the private sector, unlike in Poland. Here, a significantly larger number of public sector employees, compared with private organisations, demonstrated their intentions to change workplace. Poland’s private sector showed greater social responsibility than its public organisations, especially in the areas of public relations, customer information, service quality, and security. Beyond the previous, respondents working in private organisations of both countries distinguished themselves by displaying significantly more responsible behaviour (Table 7).

In some cases, the differences were very indistinct and statistically insignificant (Table 8). In turn, similar regularities were found as those in the Polish study sample (Table 9). In the case of Lithuania, links between the level of corporate social responsibility and how employees assessed their interrelationships could be noticed. That is, the most favourable situation was recorded in the enterprises that had declared CSR, unlike in enterprises that did not seek such status, while the organisations that were currently in the process occupied an intermediate position. Moreover, similar trends favourable to employees remained in the task assignment area, but no statistically significant differences were recorded in the areas of leadership and work environment. This may indicate the existence of a well-established leadership style and attitude towards the work environment that is not influenced by CSR. On the other hand, although a significantly greater focus on external stakeholders came to prominence in CSR-oriented organisations, trends of the very employees’ attitudes showed the opposite perspective. Employees of organisations that have claimed CSR and are seeking CSR tend to transfer concern about socially responsible activity to the organisations themselves. In addition, employee relationships with customers or product users across all three levels of organisations in principal did not differ.

Problems related to employee interrelationships, tasks, work content, and assessment in all aspects were more often encountered by organisations both seeking and not seeking to become socially responsible in Poland, unlike in Lithuania. Similarly, Polish organisations not seeking to become CSR organisations were more “troubled” with the problems of work organisation and leadership as well as work environment and working conditions than Lithuanian organisations, but these types of Lithuanian organisations appeared to have more expressed aspects of the socially responsible organisation and employee behaviour than respective Polish organisations. Furthermore, CSR organisations in Poland, more often than in Lithuania, experienced problems related to employee demographics, attitudes, experienced harm, work assessment and tasks, while in Lithuania, problems of such organisations mostly pertained to the aspects of work organisation and leadership. Responsibility in the areas of environmental protection and relationships with employees was the strength of Poland’s CSR organisations, compared with CSR organisations in Lithuania. Responsibility of the employees themselves to customers in the organisations declaring social responsibility in principal did not differ, but statistically significant differences were seen in the other two groups, where respondents from Lithuania demonstrated greater responsibility (Table 10).

The data presented allow us to state that hypothesis H3 can also be confirmed.

Less than a quarter of all employees who had experienced workplace mobbing in Polish organisations were not inclined to care about the quality of services and good relationships with users of the services and products, while about a tenth followed the approach that CSR was not their responsibility, but that of the organisation. However, the Chi-square test did not show statistically significant differences. Meanwhile, in the Lithuanian sample, the area of care about services/products was the only area where no statistically significant differences were identified. In this case, there was a higher percentage of employees who were less concerned with the quality of services and products, like those opting out of CSR (Table 11). Table 11 Attitudes of Employees who Have Experienced Workplace Mobbing to CSR (*N* = 823).

One can then state that H4 was confirmed, though at a low level.

After performing the regression analysis in the case of Polish organisations (Table 12), it was found that, while the independent variables related to employee interrelationships, work organisation and management, and working environment and conditions decreased separately one at a time in succession and the other remaining variables did not change, *socially responsible* organisational behaviour improved. That is, with the decrease in almost all factors of mobbing as a psychosocial stressor (except for one–factors related to the nature of tasks, work content and assessment), the behaviour of the socially responsible organisation in the case of Polish organisations improved. While negative factors related to employee interrelationships, the nature of tasks, work content and assessment, working environment and conditions decreased separately one at a time in succession and the other remaining variables did not change, socially responsible employee behaviour improved (i.e., statistically significant reliability (difference) is found in three out of the four variables. Table 12 Relationships between Mobbing As a Psychosocial Stressor and Corporate Social Responsibility Categories in Polish Organisations (N = 413) and Lithuanian organisations (N = 410).

In turn, after performing the regression analysis in the case of Lithuanian organisations, analysing the dependent variable of socially responsible organisational behaviour, it was found that while independent variables related to employee interrelationships, the nature of tasks, work content and assessment, work organisation and management, and working environment and conditions decreased separately one at a time in succession and the other remaining variables did not change, socially responsible organisational behaviour improved. That is, when absolutely all factors of mobbing as a psychosocial stressor (as diagnosed in this study) decreased, socially responsible organisational behaviour improved in the case of Lithuanian organisations.

Analysing the second dependent variable of socially responsible employee behaviour, it was found that while independent variables related to the nature of tasks, work content and assessment decreased, socially responsible employee behaviour improved (i.e., statistically significant reliability (difference) was found in one out of the four variables).

## 5. Conclusions

Although Poland and Lithuania are neighbouring states with common historical, cultural, and religious links, our study demonstrated significant differences with regard to organisational attitudes towards CSR as well as in the aspect of the possibility for employees to experience mobbing. However, this study is important not so much for distinguishing differences, but for its novelty and significance in uncovering links between the mobbing phenomenon and CSR and employee behaviour.

The results of our study demonstrate that there may be links between organisational and employee attitudes to CSR. That is, in organisations with a lower focus on social responsibility, employees also exhibit a respectively worse attitude. On the other hand, this can also be related to management behaviour and interpersonal relationships. In the Polish public sector, for example, significantly worse interpersonal relations and negative managerial actions as well as less social responsibility were identified. This can also be related to a more negligent attitude of employees to CSR. Significantly, the less likelihood of Lithuanian public and private sector employees to change jobs can be explained by the similarity of the situation in both sectors, which means that employees do not expect to encounter a more favourable situation should they cross-over. This assumption was also confirmed by the situation in Polish private and public sectors. Here, the situation in the public sector was much worse, and employees were more likely to change jobs. This can also be related to the worse situation in the sector in terms of CSR determining employees’ greater dissatisfaction with their jobs. Of note, this study did not investigate labour market opportunities and remuneration, which could also influence employees’ intentions to change jobs; therefore, this should be clarified in another study. We also did not investigate in what sectors employees were looking for new jobs.

Trends of employee relation to CSR that showed up in the case of both countries show the existence of two speed lanes. This could be explained by the fact that CSR-oriented organisations are more focused on external stakeholders than on the even development of the idea within the organisation itself, due to which they do not acquire significant support. Interestingly, the employees themselves assume greater responsibility in the organisations that do not declare CSR, as if trying to compensate for the entities’ shortcomings. Such behaviour may be more influenced by the values formed in the society, affecting the attitude to work and relationships with customers or product users. A certain resistance factor related to change being introduced by CSR-oriented organisations in general also cannot be ruled out. Further research is required to explain the difference demonstrating less responsibility among employees of organisations orienting to and declaring CSR in relationships with service/product users and customers. In this case, more detailed fields of research, related to responses to change, value congruence, and organisational cynicism, may be useful. For example, employee cynicism comes to prominence when a discrepancy between managerial declarations and reality is noticed [78,79]. Thus, new insights would help to implement CSR-related changes in a more consistent and efficient manner. It is likely that organisations seeking CSR status pay more attention to internal processes that guarantee a more favourable situation for employees in the context of interpersonal relationships. Still, CSR organisations may be focused on external stakeholders and image retention, as shown by several studies conducted over the last decade [80,81,82,83,84].

There are several contributions to the science of our study. First, it presents the results of quantitative research related to the attitude of employees of Polish and Lithuanian organisations that implement the CSR concept on the phenomenon of workplace mobbing. In addition, it is an international approach built upon the basis of two culturally and geographically close countries and on a large sample (total of 823) of respondents. Another advantage is the fact that due to the sensitive nature of the topic; such research is rare not only in both countries, but also on an international scale. Thus, this aspect may be regarded as a significant added value of the study. Moreover, our study is significant in that it supplements the understanding of mobbing in the context of the social responsibility of entities operating in both public and private sectors. Furthermore, one should add that our research confirmed the fact that no country is free from the mobbing phenomenon. Therefore, this approach can serve to diminish the presence of mobbing and mitigate its outcome. It can also contribute to the development of a socially responsible policy that pays greater attention to internal stakeholders. In other words, the knowledge of this fact may be useful for managers, and first of all, for company owners by enabling them to take the necessary preventive actions. In addition, managers in both the public and private sector are provided with additional knowledge of the risks associated with mobbing, thus allowing them to take preventive actions. Finally, we believe that the results of our research may be very useful for the formulation of the research hypotheses in further surveys.

Our study is not free from some limitations. For example, it did not separately investigate what internal processes determine the better status of employee relationships in organisations seeking CSR, as compared with those already declaring this status. Future research in this area could better explain the reasons for these differences. Despite this fact, we strongly believe that this study presents the real situation with regard to workplace mobbing prevalence in both countries.

## Figures and Tables

**Table 1:** Psychometric Characteristics of The Questionnaire “MOB-CSR“ (*N* min. = 823; *N* max. = 823 is 823).

Categories	Subcategories		N = 113	** Explained Dispersion, %	Cronbach’s Alpha	Spearman–Brown	*** Factor Loading (L)			**** Total Item Correlation (r/itt)		
							mean	min	max	mean	min	max
Factors related to employee interrelationships	Employee communication		7	67.67	0.92	0.89	0.82	0.77	0.87	0.67	0.52	0.85
	Employee isolation		6	78.10	0.94	0.92	0.88	0.84	0.91	0.78	0.67	0.91
	Employee reputation		6	70.68	0.92	0.91	0.84	0.75	0.89	0.70	0.54	0.90
	Employee demography		7	68.97	0.93	0.91	0.83	0.79	0.88	0.69	0.57	0.88
	Employee views		3	79.83	0.87	-	0.89	0.88	0.91	0.80	0.66	0.91
	Damage experienced by employees		5	78.82	0.93	0.89	0.89	0.86	0.92	0.79	0.68	0.91
	Employees’ emotional state		14	65.37	0.96	0.94	0.81	0.73	0.86	0.65	0.40	0.86
	Employee intentions		5	82.58	0.95	0.94	0.91	0.88	0.94	0.82	0.71	0.94
Factors related to the nature of tasks, work content and assessment	Nature of tasks		7	63.51	0.90	0.88	0.79	0.57	0.88	0.62	0.31	0.86
	Work content	Requiring intellectual work	2	84.06	-	-	0.92	0.92	0.92	0.84	0.68	0.92
		Requiring physical work	2	70.15	-	-	0.84	0.84	0.84	0.69	0.40	0.86
	Work assessment		5	81.75	0.94	0.93	0.90	0.88	0.92	0.82	0.69	0.92
Factors related to work organisation and management *	Work organisation		5	73.82	0.91	0.88	0.86	0.81	0.88	0.73	0.60	0.88
	Work management		5	76.40	0.92	0.89	0.87	0.84	0.90	0.76	0.62	0.90
Factors related to physical working environment and conditions *	Working environment		5	73.73	0.91	0.90	0.86	0.83	0.90	0.73	0.60	0.89
	Working conditions		5	63.68	0.86	0.84	0.80	0.75	0.86	0.63	0.48	0.84
Socially responsible organisational behaviour	Services and their quality		6	74.56	0.93	0.90	0.86	0.83	0.88	0.74	0.63	0.88
	Customer information, health and safety		5	74.22	0.91	0.89	0.86	0.85	0.88	0.74	0.61	0.88
	Environmental responsibility		7	73.52	0.94	0.90	0.86	0.79	0.90	0.73	0.55	0.89
	Responsibility in relationships with the society		6	67.50	0.91	0.90	0.82	0.79	0.85	0.67	0.52	0.85
	Responsibility in relationships with employees		7	71.43	0.93	0.92	0.84	0.82	0.87	0.71	0.61	0.87
Socially responsible employee behaviour *	Employees’ responsibility towards customers		4	80.87	0.92	-	0.90	0.83	0.93	0.81	0.64	0.93
	Employees’ relationships with customers		3	51.08	0.75	-	0.70	0.48	0.85	0.48	0.07	0.81

**Notes:** * all statements of these categories were recoded. ** Explained dispersion percentage cannot be lower than 10 per cent. *** Minimum factor loading (L) cannot be lower than 0.3. **** The mean of the total item correlation (r/itt) cannot be lower than.

**Table 2:** Secondary Factoring Results of The Questionnaire “MOB-CSR” Categories and Subcategories (*N* min. = 823; *N* max. = 823).

Factoring in Accordance with Principal Components (1 factor model) F1 Method			Factoring in Accordance with Alpha Factoring F1 Method		
Categories and Subcategories		N = 823	Categories and Subcategories		N = 823
**Factors Related to Employee Interrelationships**					
Employee isolation		0.88	Employee isolation		0.87
Employee reputation		0.85	Employee reputation		0.82
Employee communication		0.83	Employees’ emotional state		0.79
Employees’ emotional state		0.81	Employee communication		0.79
Damage experienced by employees		0.79	Damage experienced by employees		0.76
Employee demography		0.78	Employee demography		0.75
Employee intentions		0.75	Employee views		0.70
Employee views		0.74	Employee intentions		0.70
Explained dispersion, %		64.99	Explained dispersion, %		60.11
**Factors Related to the Nature of Tasks, work Content and Assessment**					
Nature of tasks		0.90	Nature of tasks		0.91
Work assessment		0.88	Work assessment		0.81
Work content		0.74	Work content		0.55
Requiring physical work	0.73		Requiring intellectual work	0.53	
Requiring intellectual work	0.73		Requiring physical work	0.53	
Explained dispersion, %	52.66		Explained dispersion, %	35.24	
Explained dispersion, %		70.96	Explained dispersion, %		59.26
**Factors Related to Work Organisation and Management**					
Work organisation		0.94	Work management		0.87
Work management		0.94	Work organisation		0.87
Explained dispersion, %		87.85	Explained dispersion, %		75.23
**Factors Related to Physical Working Environment and Conditions**					
Working environment		0.92	Working environment		0.83
Working conditions		0.92	Working conditions		0.83
Explained dispersion, %		84.59	Explained dispersion, %		69.10
**Socially Responsible Organisational Behaviour**					
Customer information, health and safety		0.87	Customer information, health and safety		0.84
Responsibility in relationships with employees		0.87	Responsibility in relationships with employees		0.84
Responsibility in relationships with the society		0.86	Responsibility in relationships with the society		0.83
Services and their quality		0.85	Services and their quality		0.80
Environmental responsibility		0.81	Environmental responsibility		0.74
Explained dispersion, %		72.75	Explained dispersion, %		66.05
**Socially Responsible Employee Behaviour**					
Employees’ relationships with customers		0.85	Employees’ responsibility towards customers		0.67
Employees’ responsibility towards customers		0.85	Employees’ relationships with customers		0.67
Explained dispersion, %		72.81	Explained dispersion, %		45.52

**Table 3:** Secondary Factoring Results of The Questionnaire “MOB-CSR” with Regard to Two Parts (*N* min.=823; *N* max.=823 is 823).

Factoring in Accordance with Principal Components (1 factor model) F1 Method		Factoring in Accordance with Alpha Factoring F1 Method	
Parts and Subcategories	N = 823	Parts and Subcategories	N = 823
**Workplace Mobbing As a Psychosocial Stressor**			
Employee isolation	0.84	Employee isolation	0.81
Employees’ emotional state	0.82	Employees’ emotional state	0.81
Employee reputation	0.81	Work assessment	0.78
Employee communication	0.78	Employee reputation	0.78
Damage experienced by employees	0.77	Employee intentions	0.75
Employee intentions	0.77	Damage experienced by employees	0.75
Work assessment	0.77	Employee communication	0.75
Nature of tasks	0.72	Nature of tasks	0.73
Employee demography	0.71	Employee demography	0.68
Work management	0.67	Work management	0.65
Employee views	0.65	Employee views	0.59
Work organisation	0.62	Work organisation	0.59
Working conditions	0.61	Working conditions	0.58
Working environment	0.58	Working environment	0.57
Work content	0.44	Work content	0.38
Explained dispersion, %	50.15	Explained dispersion, %	47.18
**Corporate Social Responsibility**			
Customer information, health and safety	0.87	Customer information, health and safety	0.80
Responsibility in relationships with employees	0.87	Responsibility in relationships with employees	0.79
Responsibility in relationships with the society	0.85	Services and their quality	0.77
Services and their quality	0.85	Responsibility in relationships with the society	0.77
Environmental responsibility	0.79	Environmental responsibility	0.70
Employees’ responsibility towards customers	0.54	Employees’ responsibility towards customers	0.44
Employees’ relationships with customers	0.45	Employees’ relationships with customers	0.39
Explained dispersion, %	54.12	Explained dispersion, %	43.73

**Table 4:** Characteristics of Respondents and The Organisations that They Represent.

Characteristics		Lithuania		Poland		In the Overall Survey Sample (LT and PL)	
		Quantity	%	Quantity	%	Quantity	%
Characteristics of the organisations							
Sector	Private sector	197	48.0	204	49.4	401	48.7
	Public sector	213	52.0	209	50.6	422	51.3
CSR	Seeks to become socially responsible	93	22.7	153	37.0	246	29.9
	Is socially responsible	244	59.5	174	42.1	418	50.8
	Does not seek to become socially responsible	73	17.8	86	20.9	159	19.3
Size of the organisation by the number of employees	Up to 10 employees (very small)	85	20.7	87	21.1	172	20.9
	More than 10 but less than 50 employees (small)	161	39.3	133	32.2	294	35.7
	From 50 to 250 employees (medium-sized)	100	24.4	122	29.5	222	27.0
	Over 250 employees (large)	64	15.6	71	17.2	135	16.4
Characteristics of respondents							
Gender	Male	154	37.6	215	52.1	369	44.8
	Female	256	62.4	198	47.9	454	55.2
Age	18–25	190	46.3	55	13.3	245	29.7
	26–30	62	15.1	68	16.5	130	15.8
	31–35	39	9.5	62	15.0	101	12.3
	36–40	25	6.1	85	20.5	110	13.4
	41–45	29	7.1	78	18.9	107	13.0
	46–50	27	6.6	37	9.0	64	7.8
	51–60	27	6.6	21	5.1	48	5.8
	61 and over	11	2.7	7	1.7	18	2.2
Education	Higher university (Bachelor: university, institute, academy)	208	50.7	112	27.1	320	38.9
	Higher non-university (professional Bachelor: college)	80	19.5	55	13.3	135	16.4
	Unfinished higher educational institution	58	14.1	36	8.7	94	11.4
	Upper secondary	19	4.6	43	10.4	62	7.5
	Vocational	18	4.4	79	19.1	97	11.8
	Secondary	25	6.1	79	19.1	104	12.6
	Primary	2	0.6	9	2.3	11	1.4
Seniority	Up to 1 year	58	14.1	22	5.3	80	9.7
	From 1 to 3 years	103	25.1	73	17.7	176	21.4
	From 4 to 7 years	83	20.2	74	17.9	157	19.1
	From 8 to 10 years	35	8.6	61	14.8	96	11.7
	From 11 to 15 years	37	9.0	60	14.5	97	11.8
	From 16 to 20 years	34	8.4	61	14.8	95	11.5
	From 21 years and more	60	14.6	62	15.0	122	14.8
Position in the organisation	Top-level manager	15	3.7	41	9.9	56	6.8
	Middle-level manager	57	13.9	46	11.1	103	12.5
	Low-level manager	42	10.2	34	8.3	76	9.3
	Ordinary employee (does not have subordinates)	262	63.9	176	42.6	438	53.2
	Worker (the person doing physical work)	34	8.3	116	28.1	150	18.2
Specificity of work	Provision of services, I directly communicate with customers, interested persons	310	75.6	242	58.6	552	67.1
	I do technical, physical work	100	24.4	171	41.4	271	32.9
Marital status	Single	116	28.3	107	25.9	223	27.1
	Married	125	30.5	191	46.2	316	38.4
	Divorced	35	8.5	38	9.3	73	8.9
	I live with my partner	134	32.7	77	18.6	211	25.6
	Total:	410	49.8	413	50.2	823	100

**Table 5:** The Situation of Private and Public Sector with Regard to Workplace Mobbing and CSR (LT, *N* = 410).

Categories	Subcategories	Lithuania, N = 410						
		Private Sector, N = 197	Public Sector, N = 213	Private Sector, N = 197	Public Sector, N = 213	Mann–Whitney U Test Results		
		Median		Rank Averages		U	Z	P
Factors related to employee interrelationships	Employee communication	1.14	1.14	198.7	211.8	19,633.0	−1.181	0.237
	Employee isolation	1.00	1.00	202.8	208.0	20,442.5	−0.484	0.628
	Employee reputation	1.33	1.50	194.3	215.9	18,771.5	−1.897	0.058
	Employee demography	1.00	1.00	206.2	204.8	20,837.0	−0.137	0.891
	Employee views	1.00	1.00	203.0	207.8	20,484.5	−0.503	0.615
	Damage experienced by employees	1.00	1.00	203.3	207.5	20,546.5	−0.395	0.692
	Employees’ emotional state	1.79	1.93	194.1	216.0	18,738.0	−1.879	0.060
	Employee intentions	2.00	2.00	207.2	204.0	20,652.5	−0.281	0.779
Factors related to the nature of tasks, work content and assessment	Nature of tasks	1.71	1.86	201.1	209.6	20,107.0	−0.731	0.465
	Work content	3.25	3.00	214.3	197.4	19,253.0	−1.452	0.146
	Work assessment	1.80	2.00	202.2	208.5	20,337.5	−0.544	0.586
Factors related to work organisation and management	Work organisation	2.00	2.40	194.5	215.7	18,818.5	−1.814	0.070
	Work management	2.20	2.40	198.9	211.6	19,671.0	−1.102	0.271
Factors related to physical working environment and conditions	Working environment	2.00	2.20	199.4	211.1	19,785.0	−1.003	0.316
	Working conditions	2.60	2.40	209.3	202.0	20,234.0	−0.625	0.532
Socially responsible organisational behaviour	Services and their quality	4.00	3.83	204.3	206.6	20,739.0	−0.203	0.839
	Customer information, health and safety	3.80	3.80	206.4	204.7	20,801.5	−0.150	0.880
	Environmental responsibility	3.14	3.29	201.1	209.6	20,110.5	−0.727	0.467
	Responsibility in relationships with greater society	3.50	3.67	197.8	212.7	19,457.5	−1.275	0.202
	Responsibility in relationships with employees	3.43	3.43	205.8	205.3	20,929.5	−0.043	0.966
Socially responsible employee behaviour	Employees’ responsibility towards customers	4.50	4.25	212.2	199.3	19,658.5	−1.143	0.253
	Employees’ relationships with customers	3.33	3.67	200.9	209.7	20,077.0	−0.763	0.446

**Table 6:** The Situation of Private and Public Sector with Regard to Workplace Mobbing and CSR (PL, *N* = 413).

Categories	Subcategories	Poland, N = 413						
		Private sector, N = 204	Public sector, N = 209	Private sector, N = 204	Public sector, N = 209	Mann-Whitney U test results		
		Median		Rank Averages		U	Z	P
Factors related to employee interrelationships	Employee communication	1.43	1.86	191.0	222.6	18,050.5	−2.750	0.006 **
	Employee isolation	1.50	2.00	194.1	219.6	18,685.5	−2.219	0.026 *
	Employee reputation	1.83	2.00	203.2	210.7	20,541.0	−0.650	0.516
	Employee demography	1.29	2.00	184.6	228.8	16,757.5	−3.860	0.0001 **
	Employee views	1.33	2.00	194.4	219.3	18,742.5	−2.222	0.026 *
	Damage experienced by employees	1.30	2.00	181.6	231.8	16,129.5	−4.398	0.0001 **
	Employees’ emotional state	2,18	2,64	196,2	217,5	19,121,0	−1.814	0.070
	Employee intentions	2,00	2,80	187,3	226,2	17,295,5	−3.335	0.001 **
Factors related to the nature of tasks, work content and assessment	Nature of tasks	2,14	2,86	182,1	231,3	16,236,5	−4.196	0.0001 **
	Work content	3,00	3,50	186,8	226,8	17,188,5	−3.431	0.001 **
	Work assessment	2,20	3,20	183,5	229,9	16,531,0	−3.961	0.0001 **
Factors related to work organisation and management	Work organisation	2.20	2.60	194.5	219.2	18,773.5	−2.107	0.035 *
	Work management	2.20	2.60	201.3	212.6	20,150.0	−0.969	0.332
Factors related to physical working environment and conditions	Working environment	2.00	2.60	185.3	228.2	16,896.5	−3.672	0.001 **
	Working conditions	2.40	2.80	199.8	214.0	19,847.5	−1.216	0.224
Socially responsible organisational behaviour	Services and their quality	4.00	3.67	235.5	179.1	15,494.5	−4.825	0.0001 **
	Customer information, health and safety	4.00	3.40	232.9	181.7	16,025.0	−4.384	0.0001 **
	Environmental responsibility	3.29	3.00	213.6	200.6	19,981.0	−1.105	0.269
	Responsibility in relationships with greater society	3.33	3.17	217.5	196.7	19,167.5	−1.778	0.075
	Responsibility in relationships with employees	3.43	3.29	215.8	198.4	19,531.0	−1.476	0.140
Socially responsible employee behaviour	Employees’ responsibility towards customers	4.00	4.00	226.9	187.6	17,255.5	−3.397	0.001 **
	Employees’ relationships with customers	3.67	3.33	223.5	190.9	17,948.0	−2.803	0.005 **

**Notes:** * all statements of these categories were recoded. ** Explained dispersion percentage cannot be lower than 10 per cent.

**Table 7:** Problems of Employee Interrelationships (LT, PL: *N* = 823).

Categories	Subcategories	Private Sector, N = 401			Public Sector, N = 422		
		Lithuania, N = 197	Poland, N = 204	Level of Statistical Significance, p	Lithuania, N = 213	Poland, N = 209	Level of Statistical Significance, p *
		Median			Median		
Factors related to employee interrelationships	Employee communication	1.14	1.43	0.004 **	1.14	1.86	0.0001 **
	Employee isolation	1.00	1.50	0.001 **	1.00	2.00	0.0001 **
	Employee reputation	1.33	1.83	0.002 **	1.50	2.00	0.023 *
	Employee demography	1.00	1.29	0.0001 **	1.00	2.00	0.0001 **
	Employee views	1.00	1.33	0.0001 **	1.00	2.00	0.0001 **
	Damage experienced by employees	1.00	1.30	0.040 *	1.00	2.00	0.0001 **
	Employees’ emotional state	1.79	2.18	0.0001 **	1.93	2.64	0.001 **
	Employee intentions	2.00	2.00	0.121	2.00	2.80	0.0001 **
Factors related to the nature of tasks, work content and assessment	Nature of tasks	1.71	2.14	0.0001 **	1.86	2.86	0.0001 **
	Work content	3.25	3.00	0.636	3.00	3.50	0.0001 **
	Work assessment	1.80	2.20	0.0001 **	2.00	3.20	0.0001 **
Factors related to work organisation and management	Work organisation	2.00	2.20	0.606	2.40	2.60	0.251
	Work management	2.20	2.20	0.254	2.40	2.60	0.208
Factors related to physical working environment and conditions	Working environment	2.00	2.00	0.547	2.20	2.60	0.024 *
	Working conditions	2.60	2.40	0.408	2.40	2.80	0.395
Socially responsible organisation’s behaviour	Services and their quality	4.00	4.00	0.033 *	3.83	3.67	0.002 **
	Customer information, health and safety	3.80	4.00	0.311	3.80	3.40	0.001 **
	Environmental responsibility	3.14	3.29	0.076	3.29	3.00	0.923
	Responsibility in relationships with the society	3.50	3.33	0.599	3.67	3.17	0.001 **
	Responsibility in relationships with employees	3.43	3.43	0.808	3.43	3.29	0.151
Socially responsible employee’s behaviour	Employees’ responsibility towards customers	4.50	4.00	0.002 **	4.25	4.00	0.0001 **
	Employees’ relationships with customers	3.33	3.67	0.063	3.67	3.33	0.066

* Mann–Whitney U test was used. ** Explained dispersion percentage cannot be lower than 10 per cent.

**Table 8:** Workplace Mobbing with Regard to CSR (LT, *N* = 410).

Categories	Subcategories	Lithuania, N = 410							
		Seeks to become CSR, N = 93	Is CSR, N = 244	Does Not Seek to Become CSR, N = 73	Seeks to Become CSR, N = 93	Is CSR, N = 244	Does Not Seek to Become CSR, N = 73	Kruskal–Wallis H Test Results	
		Median			Rank Averages			*χ*2	*P*
Factors related to employee interrelationships	Employee communication	1.19	1.14	1.43	209.5	194.8	236.2	7.729	0.021 *
	Employee isolation	1.17	1.00	1.33	207.3	197.5	230.1	4.973	0.083
	Employee reputation	1.33	1.43	1.83	197.2	202.5	226.2	3.002	0.223
	Employee demography	1.00	1.00	1.14	213.0	194.4	232.9	8.457	0.015 *
	Employee views	1.00	0.67	1.33	202.7	196.8	238.3	10.317	0.006 **
	Damage experienced by employees	1.16	1.00	1.40	207.4	194.0	241.6	10.832	0.004 **
	Employees’ emotional state	1.71	1.79	2.00	195.6	198.4	241.8	8.446	0.015 *
	Employee intentions	1.60	2.00	2.20	196.8	199.1	238.0	7.083	0.029 *
Factors related to the nature of tasks, work content and assessment	Nature of tasks	2.00	1.71	2.14	224.8	187.2	242.1	15.338	0.001 **
	Work content	3.00	3.00	3.25	192.7	203.1	229.7	4.306	0.116
	Work assessment	1.80	1.80	2.20	195.1	195.7	251.6	13.852	0.001 **
Factors related to work organisation and management	Work organisation	2.20	2.20	2.20	199.5	205.4	213.5	0.576	0.750
	Work management	2.20	2.20	2.40	204.7	197.6	232.9	5.073	0.079
Factors related to physical working environment and conditions	Working environment	2.20	2.20	2.20	220.2	197.5	213.6	2.915	0.233
	Working conditions	2.40	2.40	2.80	198.9	198.9	235.9	5.885	0.053
Socially responsible organisation’s behaviour	Services and their quality	4.00	4.00	3.67	205.1	215.2	173.5	7.030	0.030 *
	Customer information, health and safety	3.80	4.00	3.60	204.9	215.1	174.1	6.836	0.033 *
	Environmental responsibility	3.00	3.29	3.00	189.2	219.6	179.2	8.824	0.012 *
	Responsibility in relationships with the society	3.50	3.67	3.00	195.4	223.6	157.8	18.315	0.001 **
	Responsibility in relationships with employees	3.29	3.57	3.00	193.9	220.0	171.8	10.489	0.005 **
Socially responsible employee’s behaviour	Employees’ responsibility towards customers	4.50	4.50	4.00	210.7	213.0	173.8	6.836	0.033 *
	Employees’ relationships with customers	3.33	3.67	3.33	201.8	215.2	178.0	5.789	0.055

**Notes:** * all statements of these categories were recoded. ** Explained dispersion percentage cannot be lower than 10 per cent.

**Table 9:** Workplace Mobbing with Regard to CSR (PL, *N* = 413).

Categories	Subcategories	Poland, N = 413							
		Seeks to Become CSR, N = 153	Is CSR, N = 174	Does Not Seek to Become CSR, N = 86	Seeks to Become CSR, N = 153	Is CSR, N = 174	Does Not Seek to Become CSR, N = 86	Kruskal–Wallis H Test Results	
		Median			Rank averages			*χ*2	*P*
Factors related to employee interrelationships	Employee communication	1.86	1.14	2.36	212.5	165.7	280.8	56.283	0.0001 **
	Employee isolation	2.00	1.17	2.50	216.7	157.6	289.7	75.297	0.0001 **
	Employee reputation	2.00	1.50	2.50	208.4	166.9	285.6	58.591	0.0001 **
	Employee demography	1.57	1.21	2.29	215.5	168.3	270.1	45.393	0.0001 **
	Employee views	2.00	1.00	2.33	221.9	165.3	264.9	47.950	0.0001 **
	Damage experienced by employees	2.00	1.40	2.40	209.0	174.0	270.1	39.460	0.0001 **
	Employees’ emotional state	2.50	2.00	3.00	209.8	163.4	290.3	65.364	0.0001 **
	Employee intentions	2.60	2.00	3.30	206.1	168.2	287.1	57.669	0.0001 **
Factors related to the nature of tasks, work content and assessment	Nature of tasks	2.57	2.00	3.14	206.6	173.8	274.9	41.379	0.0001 **
	Work content	3.25	3.00	3.50	207.1	188.1	245.2	13.369	0.001 **
	Work assessment	3.00	2.00	3.40	211.2	174.1	266.2	34.803	0.0001 **
Factors related to work organisation and management	Work organisation	2.60	2.00	3.00	206.9	172.4	277.1	44.635	0.0001 **
	Work management	2.60	2.00	3.10	205.6	169.4	285.6	55.245	0.0001 **
Factors related to physical working environment and conditions	Working environment	2.20	2.00	3.10	202.4	174.5	281.0	46.908	0.0001 **
	Working conditions	2.60	2.20	3.20	201.4	176.2	279.4	43.842	0.0001 **
Socially responsible organisation’s behaviour	Services and their quality	3.83	4.00	3.00	209.3	241.0	134.3	46.537	0.0001 **
	Customer information, health and safety	3.80	4.00	3.00	205.6	245.5	131.5	52.944	0.0001 **
	Environmental responsibility	3.14	3.57	2.71	210.8	243.0	127.5	54.326	0.0001 **
	Responsibility in relationships with the society	3.33	3.67	2.67	210.0	249.1	116.5	71.489	0.0001 **
	Responsibility in relationships with employees	3.29	3.71	2.57	208.2	252.1	113.7	77.679	0.0001 **
Socially responsible employee’s behaviour	Employees’ responsibility towards customers	4.00	4.25	3.50	195.6	252.7	134.7	60.115	0.0001 **
	Employees’ relationships with customers	3.33	3.67	3.00	206.0	229.2	163.9	17.526	0.001 **

**Notes:** * all statements of these categories were recoded. ** Explained dispersion percentage cannot be lower than 10 per cent.

**Table 10:** Workplace Mobbing with Regard to CSR (LT, PL, *N* = 823).

Categories	Subcategories	Seeks to Become CSR, N = 246			Is CSR, N = 418			Does Not Seek to Become CSR, N = 159		
		LT, N = 93	PL, N = 153	Level of Statistical Significance, p	LT, N = 244	PL, N = 174	Level of Statistical Significance, p	LT, N = 73	PL, N = 86	Level of Statistical Significance, p **
		Median			Median			Median		
Factors related to employee interrelationships	Employee communication	1.19	1.86	0.002 **	1.14	1.14	0.109	1.43	2.36	0.0001 **
	Employee isolation	1.17	2.00	0.001 **	1.00	1.17	0.202	1.33	2.50	0.0001 **
	Employee reputation	1.33	2.00	0.012 *	1.43	1.50	0.834	1.83	2.50	0.0001 **
	Employee demography	1.00	1.57	0.0001 **	1.00	1.21	0.0001 **	1.14	2.29	0.0001 **
	Employee views	1.00	2.00	0.0001 **	0.67	1.00	0.001 **	1.33	2.33	0.0001 **
	Damage experienced by employees	1.16	2.00	0.007 **	1.00	1.40	0.008 **	1.40	2.40	0.0001 **
	Employees’ emotional state	1.71	2.50	0.0001 **	1.79	2.00	0.277	2.00	3.00	0.0001 **
	Employee intentions	1.60	2.60	0.003 **	2.00	2.00	0.330	2.20	3.30	0.0001 **
Factors related to the nature of tasks, work content and assessment	Nature of tasks	2.00	2.57	0.0001 **	1.71	2.00	0.0001 **	2.14	3.14	0.0001 **
	Work content	3.00	3.25	0.015 *	3.00	3.00	0.648	3.25	3.50	0.015 *
	Work assessment	1.80	3.00	0.0001 **	1.80	2.00	0.001 **	2.20	3.40	0.0001 **
Factors related to work organisation and management	Work organisation	2.20	2.60	0.252	2.20	2.00	0.034 *	2.20	3.00	0.001 **
	Work management	2.20	2.60	0.363	2.20	2.00	0.260	2.40	3.10	0.001 **
Factors related to physical working environment and conditions	Working environment	2.20	2.20	0.633	2.20	2.00	0.162	2.20	3.10	0.0001 **
	Working conditions	2.40	2.60	0.905	2.40	2.20	0.075	2.80	3.20	0.015 *
Socially responsible organisation’s behaviour	Services and their quality	4.00	3.83	0.950	4.00	4.00	0.121	3.67	3.00	0.018 *
	Customer information, health and safety	3.80	3.80	0.334	4.00	4.00	0.216	3.60	3.00	0.001 **
	Environmental responsibility	3.00	3.14	0.055	3.29	3.57	0.016 *	3.00	2.71	0.207
	Responsibility in relationships with the society	3.50	3.33	0.270	3.67	3.67	0.800	3.00	2.67	0.002 **
	Responsibility in relationships with employees	3.29	3.29	0.870	3.57	3.71	0.039 *	3.00	2.57	0.003 **
Socially responsible employee’s behaviour	Employees’ responsibility towards customers	4.50	4.00	0.0001 **	4.50	4.25	0.166	4.00	3.50	0.0001 **
	Employees’ relationships with customers	3.33	3.33	0.929	3.67	3.67	0.217	3.33	3.00	0.407

**Note:** * statistical significance level α = 0.05; ** statistical significance level α = 0.01.

**Table 11:** Attitudes of employees who have experienced workplace mobbing to CSR (*N* = 823).

Workplace Mobbing		Care about Quality of Services/Products				Chi-Square Test Results		Care about Users of Services/Products				Chi-Square Test Results		React to Claims of Users of Services/Products				Chi-Square Test Results		Do Not Opt Out of Responsibility				Chi-Square Test Results	
		Agree		Disagree				Agree		Disagree				Agree		Disagree				Agree		Disagree			
		N	%	N	%	χ2	p	N	%	N	%	χ2	p	N	%	N	%	χ2	p	N	%	N	%	χ3	P
LT	Did not experience	360	92.5	17	81.0	3.618	0.057	355	93.2	22	75.9	10.915	0.001 **	352	92.9	25	80.6	5.792	0.016 *	342	93.7	35	77.8	13.720	0.001 **
	Experienced	29	7.5	4	19.0			26	6.8	7	24.1			27	7.1	6	19.4			23	6.3	10	22.2		
PL	Did not experience	327	89.6	39	81.3	2.925	0.087	337	89.2	29	82.9	1.259	0.262	330	89.2	36	83.7	1.142	0.285	291	88.7	75	88.2	0.016	0.900
	Experienced	38	10.4	9	18.8			41	10.8	6	17.1			40	10.8	7	16.3			37	11.3	10	11.8		

**Note:** * statistical significance level α = 0.05; ** statistical significance level α = 0.01.

**Table 12:** Relationships between mobbing as a psychosocial stressor and corporate social responsibility categories in Polish organisations (N = 413) and Lithuanian organisations (*N* = 410).

	Dependent Variable—FOSB				Dependent Variable—FESB			
	R	R^2^	R^2^ Revised	Reliability	R	R^2^	R^2^ Revised	Reliability
Poland	0.825	0.681	0.678	0.000	0.562	0.316	0.309	0.000
Independent Variable	Non-Standardised Beta Coefficient	Standardised Beta Coefficient	t	ANOVA Reliability	Non-Standardised Beta Coefficient	Standardised Beta Coefficient	t	ANOVA Reliability
(Constant)	5.530		66.717	0.000	5.194		43.256	0.000
FEIR	−0.304	−0.289	−7.309	0.000	−0.218	−0.209	−3.605	0.000
FNCA	−0.024	−0.028	−0.828	0.408	−0.214	−0.254	−5.110	0.000
FWOM	−0.197	−0.226	−5.353	0.000	0.069	0.079	1.287	0.199
FPEC	−0.372	−0.424	−10.251	0.000	−0.259	−0.299	−4.934	0.000
Lithuania	0.758	0.575	0.571	0.000	0.242	0.059	0.049	0.000
**Independent Variable**	**Non-Standardised Beta** **Coefficient**	**Standardised** **Beta** **Coefficient**	**t**	**ANOVA Reliability**	**Non-Standardised Beta** **Coefficient**	**Standardised** **Beta** **Coefficient**	**t**	**ANOVA Reliability**
(Constant)	5.226		44.539	0.000	4.282		27.558	0.000
FEIR	−0.198	−0.155	−2.932	0.004	−0.147	−0.129	−1.647	0.100
FNCA	−0.155	−0.121	−2.489	0.013	−0.183	−0.161	−2.216	0.027
FWOM	−0.282	−0.301	−5.806	0.000	0.101	0.122	1.577	0.116
FPEC	−0.457	−0.462	−9.280	0.000	−0.017	−0.019	−0.260	0.795

**Note:** R = set correlation coefficient; R^2^ = aggregate coefficient of certainty (coefficient of determination); F = observed value of Fisher’s statistics. FEIR—factors related to employee interrelationship; FNCA—factors related to the nature of tasks, work content and assessment; FWOM—factors related to work organisation and management; FPEC—factors related to physical working environment and conditions; FOSB—factors related to behaviour of socially responsible organisation; andFESB—factors related to behaviour of socially responsible employee. Regression equations: **Poland** FOSB = 5.530–0.304 * FEIR1–0.197 * FWOM3–0.372 * FPEC4. FESB = 5.194–0.218 * FEIR1–0.214 * FNCA2–0.259 * FPEC4. **Lithuania** FOSB = 5.226–0.198 * FEIR1–0.155 * FNCA2–0.282 * FWOM3–0.457 * FPEC4. FESB = 4.282–0.183 * FNCA2.
